# Test-Retest Reliability of Resting Brain Small-World Network Properties across Different Data Processing and Modeling Strategies

**DOI:** 10.3390/brainsci13050825

**Published:** 2023-05-19

**Authors:** Qianying Wu, Hui Lei, Tianxin Mao, Yao Deng, Xiaocui Zhang, Yali Jiang, Xue Zhong, John A. Detre, Jianghong Liu, Hengyi Rao

**Affiliations:** 1 Key Laboratory of Brain-Machine Intelligence for Information Behavior (Ministry of Education and Shanghai), School of Business and Management, Shanghai International Studies University, Shanghai 201613, China; 2 Department of Neurology, University of Pennsylvania, Philadelphia, PA 19104, USA; 3 School of Life Sciences, University of Science and Technology of China, Hefei 230026, China; 4 College of Education, Hunan Agricultural University, Changsha 410127, China; 5 Medical Psychological Center, The Second Xiangya Hospital, Central South University, Changsha 410017, China; 6 Medical Psychological Institute, Central South University, Changsha 410017, China; 7 National Clinical Research Center for Mental Disorders, Changsha 410011, China; 8 Department of Family and Community Health, School of Nursing, University of Pennsylvania, Philadelphia, PA 19104, USA

**Keywords:** resting-state fMRI, test-retest reliability, graph theoretical modeling, small-world network (SWN), intra-class correlation coefficient (ICC)

## Abstract

Resting-state functional magnetic resonance imaging (fMRI) with graph theoretical modeling has been increasingly applied for assessing whole brain network topological organization, yet its reproducibility remains controversial. In this study, we acquired three repeated resting-state fMRI scans from 16 healthy controls during a strictly controlled in-laboratory study and examined the test-retest reliability of seven global and three nodal brain network metrics using different data processing and modeling strategies. Among the global network metrics, the characteristic path length exhibited the highest reliability, whereas the network small-worldness performed the poorest. Nodal efficiency was the most reliable nodal metric, whereas betweenness centrality showed the lowest reliability. Weighted global network metrics provided better reliability than binary metrics, and reliability from the AAL90 atlas outweighed those from the Power264 parcellation. Although global signal regression had no consistent effects on the reliability of global network metrics, it slightly impaired the reliability of nodal metrics. These findings provide important implications for the future utility of graph theoretical modeling in brain network analyses.

## 1. Introduction

Resting-state functional magnetic resonance imaging (rs-fMRI) has proven to be a powerful tool for examining spontaneous fluctuations in brain activities. The spontaneous activity of the resting brain, often referred to as the intrinsic baseline brain function, likely represents a ‘physiologic, functionally significant state of the brain’. In the resting state, task-evoked energy consumption appears to be less than 5% of that during basal metabolism [1,2]. Functional connectivity has been widely applied to characterize resting-state brain activities. It is defined as ‘the temporal correlation between neurophysiological measurements made in different brain areas’ [3] (p. 6) and reflects the level of information processing and transportation between anatomically separated brain regions [4].

With the concept of functional connectivity, the brain can be viewed as a highly connected network that consistently maintains a balance between the wiring cost and processing efficiency [5]. Such functional connectivity networks have been further modeled using graph theory. In graph theory, a graph consists of two fundamental elements: node and edge. Mapping onto the rs-fMRI data, nodes are typically defined by regions of interest (ROI) or voxels, and edges are represented by functional connectivity strengths. 

Small-world architecture is an important graph-theoretical property of the brain network [6]. Consider two extreme situations: a regular network and a random network. The former is highly ordered, in which every node is connected to, and only connected to, its nearest neighbors (i.e., no probabilistic edge). The latter is entirely random, in which every pair of nodes can be connected independently with equal probabilities. A small-world network is in the middle and the connections are probabilistic; however, the probability follows some rules. For example, two nodes far away from each other are usually not directly connected by a single edge, whereas one can easily reach the other through a small number of steps (i.e., intermediate nodes and edges) [7]. Such properties of a small-world network are quantified by several metrics, such as the clustering coefficient and characteristic path length [8]. The brain network has been demonstrated to have a high clustering coefficient and short characteristic path length, which is crucial for maintaining efficient information segregation and integration [9]. Given such great importance of the brain’s small-world properties, they have been extensively studied and applied as biomarkers to identify various psychopathologies [10,11,12,13,14,15].

Despite the explosive application of the resting-state BOLD fMRI and graph theory, some of the results may suffer from low test-retest reliability, as measured by intra-class correlation (ICC). A review of 15 fMRI studies found that the mean voxel- or ROI-based ICC across all the studies ranged from 0.16 to 0.88, with an average of 0.50 [16]. Similarly, a recent meta-analysis reported that the overall reliability of functional connectivity is poor (average ICC = 0.29) [17]. The ICC of small-world metrics was unsatisfying as well (e.g., clustering coefficient, characteristic path length, small-worldness, etc.), and many of them hardly exceeded 0.6 [18,19,20,21].

Such poor test-retest reliabilities of the graph-theoretical measures in the rs-fMRI data hamper their application in clinical settings; therefore, it is essential to explore the optimal strategy for network analysis. An important source of inconsistency may derive from the methods of data processing and network construction. When defining the nodes, different ROI parcellations can lead to different interpretations. For example, some atlases are generated by structural separations of the brain areas (e.g., Automated-Anatomical Labeling (AAL), Havard-Oxford atlas (HOA), Brainnetome atlas (BN)), while many others are based on functional homogeneities (i.e., co-activations) (e.g., Power 264, funROI, DOS) [22,23,24,25,26]. Previous studies comparing ICC across atlases suggested that the HOA is generally more reliable than the AAL and DOS atlas [20]; and that finer parcellations, with more regions, could produce more reliable clustering coefficients and local efficiency [27]. It is also important to consider the trade-off between greater functional homogeneity (more ROIs) and better anatomical interpretability (fewer ROIs) [28].

There is further controversy regarding the definition of edge. The primary issue is whether to apply the global signal regression (GSR) during fMRI preprocessing. Global signal regression refers to the process of removing the average time series across voxels (i.e., the global signal) from each voxel signal to improve the signal-to-noise ratio [29]. However, GSR can produce spurious negative correlations, and global signals may correlate with the experimental manipulation [30]. Therefore, it is not appropriate to interpret them entirely as nuisance variables [31,32]. It is so far not clear whether GSR has a positive or negative effect on TRT reliability. Andellini et al., (2015) examined the ICC of five micro-level graph theoretical metrics (degree, clustering coefficient, local efficiency, global efficiency, and assortativity) and found that global signal regression would decrease the reliability of all the metrics [18]. On the contrary, Braun et al. (2012) reported that the effect of global signal regression depends on the exact metric and network density; however, in most cases, the ICC improved after GSR [19].

Another issue regarding the choice of the edge is the use of binary versus weighted edges. Most studies prefer the binary network because it is straightforward to model and interpret. Despite the difficulty in modeling and interpretation, the weighted network contains more information and presents a more detailed picture of the brain [33]. Previous studies have compared the TRT reliability between these two modeling strategies. For example, Andellini et al., (2015) reported that the ICC of graph theoretical metrics was not significantly improved (although slightly improved) in the weighted network modeling compared to the binary network modeling [18]. However, Xiang et al., (2019) found that weighted modeling significantly benefits the TRT reliability clustering coefficient, shortest path length, and local and global efficiency [21].

In addition to the choice of data processing and network construction methods, variations and confounding factors during subject recruitment and data collection also impair TRT reliability. For example, subjects with sleep disorders or substance abuse need to be screened and excluded, since the organization of functional brain networks of those patients significantly differs from that of healthy controls [34,35,36,37]. Moreover, resting-state data collected with eyes closed were less reliable than those with eyes open [38,39], likely caused by drowsiness when eyes were closed [17]. In addition, the consistency of scanners and sites is a relevant factor: inter-site and inter-scanner differences decrease the TRT reliability of the temporal signal-to-noise ratio (tSNR) and functional connectivity [40,41]. Finally, the brain status may vary if the scans take place at different times of the day or different days of the week; the volume of the brain, glucose metabolism, regional cerebral blood flow, and rs-fMRI-based functional connectivity all fluctuate, possibly due to the effects of circadian rhythm on the brain [42,43,44]. 

Previous investigations on resting-state test-retest reliability were primarily based on fMRI data from existing datasets (e.g., [20,21,27,45,46]). However, the participants’ health conditions were not well monitored, and many data collection details were not available. Moreover, there is no current consensus regarding data processing and network construction methods, and few studies have been able to consider multiple aspects of the analysis simultaneously. To address these important issues, we conducted a very strictly controlled experiment in which participants remained in the laboratory for five consecutive days (four nights). They were continuously monitored by research and hospital staff and were allowed to sleep for 8–9 h. All the scan sessions took place using the same scanner at the same time of the day. To further rule out potential confounders, caffeine, alcohol, tobacco, and medications were not permitted. After ruling out potential confounders in the data collection, we aimed to examine the optimal combination of strategies that would yield the highest test-retest reliability in the brain network’s small-world properties. Two nodal (degree and betweenness centrality) and five global metrics (clustering coefficient, characteristic path length, small-worldness, global efficiency, and local efficiency) were applied to characterize the overall topology of the network. Brain networks were constructed using eight different processing strategies, and the ICC was calculated to reflect the TRT reliability. We sought to answer the following three questions: (1) Which graph-theoretical measure of the brain network is the most reliable? (2) Is it necessary to apply global signal regression and use a weighted network during modeling? (3) Is AAL-90 a reliable parcellation scheme for brain network modeling?

## 2. Materials and Methods

### 2.1. Subjects

Sixteen healthy adults (8 females, mean age = 35.4 ± 9.5 yrs) were recruited as the control subjects in a very strictly controlled in-laboratory sleep study [47,48]. 

Upon recruitment, participants reported habitual sleep duration between 6.5 h–8.5 h, bedtime between 22:00–00:00, and awakenings between 06:00–09:00. Prior to the in-laboratory study, their reports were confirmed using approximately one week of wrist actigraphy. Participants assessed by questionnaire who reported habitual napping, sleep disturbances, and extreme morningness or eveningness chronotypes were excluded from the study. Screenings for acute or chronic medical and psychological conditions, as well as drug and alcohol intake, were conducted using questionnaires, physical examinations, and blood and urine tests. All participants were nonsmokers and did not participate in shift work, transmeridian travel, or irregular sleep-wake routines 60 days prior to the study. Starting one week before the end of the laboratory session, participants were not permitted to use caffeine, alcohol, tobacco, and medications (except oral contraceptives), as verified by urine screenings.

The study was approved by the Institutional Review Board (IRB) of the University of Pennsylvania (IRB ID# 811678). Informed consent was obtained before enrollment, and the subjects were compensated for their participation.

### 2.2. Experimental Design

To ensure adherence to the protocol, participants remained in the laboratory at the Clinical Translational Research Center at the Hospital of the University of Pennsylvania for 5 consecutive days (4 consecutive nights). They were behaviorally monitored by trained staff, allowed to watch television, read, play video or board games, and perform other sedentary activities, but they were not allowed to exercise or leave the laboratory.

Participants received 9 h time in bed (21:30–06:20) on day 1 to adjust to the laboratory environment and 8 h of sleep (22:30–06:30) on days 2–5. MRI scan sessions were on the morning of days 2, 3 and 5, from 7.00 a.m. to 10.00 a.m.

### 2.3. Imaging Data Acquisition and Preprocessing

Magnetic resonance imaging was conducted using a Siemens 3.0 Tesla Trio whole-body scanner (Siemens AG, Erlangen, Germany) and a standard array coil. Resting-state BOLD fMRI data were collected using the standard EPI sequence: TR = 2 s, TE = 24 ms, FOV = 220 × 220 mm^2^, matrix = 64 × 64 × 36, slice thickness = 4 mm, and inter-slice gap = 4 mm. A total of 210 images were acquired for each subject. Subjects were instructed to keep their eyes open and look at a cross fixation in the scanner. T1-weighted structural images were obtained using a standard 3D MPRAGE sequence: TR = 1.62 s, TE = 3.09 ms, FOV = 187 × 250 mm^2^, matrix size = 192 × 256, slice thickness = 5 mm, and inter-slice gap = 1 mm.

Rs-fMRI data were pre-processed and analyzed using the Data Processing Assistant for Resting-State fMRI (DPARSF V2.3_20130615; http://rfmri.org/DPARSF, accessed on 3 March 2023), which is based on Statistical Parametric Mapping software (SPM8, Wellcome Department of Cognitive Neurology, London, UK) and the REST_V1.8_130615 toolbox http://www.restfmri.net/forum/REST_V1.8, accessed on 3 March 2023) implemented in Matlab14 (MathWorks, Natick, MA, USA). The pipeline consisted of head motion correction, co-registration, smoothing with an 8 mm full-width at half-maximum (FWHM) isotropic Gaussian kernel, normalization to the standard Montreal Neurological Institute (MNI) space, and the removal of linear trends. All functional volumes were band-pass filtered (0.01 Hz < f < 0.08 Hz) in order to reduce low-frequency drift and physiological high-frequency respiratory and cardiac noise. Nuisance covariates including six head motion parameters, white matter signal, and CSF signal were regressed out. 

### 2.4. Brain Network Construction

A summary of all eight methods is in Table 1. Four of the methods were developed using a combination of two network types (binary/weighted), and with/without global signal regression, primarily using the AAL-90 atlas because AAL-90 was used in most of the previous studies [11,19,49]. Next, a comparison with the Power264 atlas, another widely used parcellation, was added in the binary/weighted networks with/without GSR. Seven global measurements were analyzed using thresholds from 0.15 to 0.35 (i.e., the proportion of strongest functional connectivity to preserve) with a 0.05 step, while three nodal measurements were analyzed based on the area under the curve (AUC) value from 0.05 to 0.50 densities.

### 2.5. Graph Theoretical Metrics

Global network metrics included mean clustering coefficient (*Cp*) and its normalized version, gamma (*γ*), characteristic path length (Lp) and its normalized version, lambda (*λ*), small-worldness (*σ*), global efficiency (Eg), and local efficiency (Eloc). Nodal metrics included degree centrality (Dc), betweenness centrality (Bc), and nodal efficiency (Ne). All the network metrics were calculated using the GRETNA toolbox [50].

Clustering coefficient (*Cp*): The clustering coefficient describes the level of closeness to form a completely connected subgraph [51]. In this study, we used the global clustering coefficient, which is equal to the average clustering coefficient of all the nodes. Gamma is the normalized *Cp* by random networks.

$$C_{p}=\frac{1}{n}\sum_{i\in N}C_{i}=\frac{1}{n}\sum_{i\in N}\frac{2t_{i}}{k_{i}{\left(k_{i}-1\right)}^{\prime }}$$

$$\gamma =C_{p}/C_{p}^{rand}$$

Characteristic path length (Lp): The characteristic path length is the mean shortest path length over all possible pairs of nodes. It helps to quantify the functional integration level [33]. Lambda is the normalized Lp by random networks.

Nodal efficiency (Ne): Nodal efficiency is defined as the average inverse shortest path length between a given node and every other node in the network [33].

Global efficiency (Eg): Global efficiency is the average nodal efficiency across all the nodes in the network. Compared to the shortest path length, it is more immediately related to parallel information transmission [8]. 

Local efficiency (Eloc): The local efficiency is proportional to the clustering coefficient and is seen as the global efficiency computed on the neighborhood of the node, also called fault tolerance [8].

Small-worldness (Sigma): Compared with random networks, small-world networks can be quantified with a larger clustering coefficient and a comparable characteristic path length, leading to a sigma σ (i.e., small-worldness) larger than one.

σ=γ/λ

Degree centrality (Dc): Degree centrality, also called degree, is the simplest measure of centrality. For binary networks, degree is the number of edges incident to the node; for weighted networks, it is the sum of weights of all the edges of the node. 

Betweenness centrality (Bc): Betweenness centrality measures the importance of a node in information communication. It is defined as the number of times the shortest path between any other node passes through a particular node.

### 2.6. Test-Retest Reliability

In order to measure the test-retest reliability of each graph-theoretical metric among three sessions, the intra-class correlation coefficient (ICC) was introduced. Specifically, as is defined and recommended in previous studies [52,53,54], we used ICC(A,1), a two-way random model, which assessed absolute agreement between measurements and considered session effects.

$$\mathrm{ICC}\left(A,1\right)=\frac{BMS-EMS}{BMS+\left(k-1\right)EMS+\left(JMS-EMS\right)\frac{k}{n}}$$

In this formula, BMS is the between-subject mean square, JMS is the between-session mean square, EMS is the mean square error, k is the number of sessions and n is the number of subjects. In this study, k = 3 and n = 16. According to Winer (1971), ICC < 0.25 is poor, 0.25–0.4 is low, 0.4–0.6 is fair, 0.6–0.75 is good, and 0.75–1.0 is excellent [55], which is what we assumed. ICCs were calculated based on SPSS (SPSS Inc. Released 2007; SPSS for Windows, Version 16.0; SPSS Inc., Chicago, IL, USA) and MATLAB 9.2 (MathWorks, Natick, MA, USA).

In addition, we calculated the within-subject coefficient of variation (CV) to account for the relative uncertainty. If the standard deviation is denoted by S, and the mean is denoted by M, then the coefficient of variation is calculated as:

(1) $$\mathrm{CV}=\frac{S}{M}$$

### 2.7. Statistical Analyses

We performed the following statistical analyses to systematically compare the TRT reliabilities of several metrics under different data processing and modeling strategies.

For global metrics, we first used a one-way repeated ANOVA to compare the effect of network thresholding on the ICC for all the metrics under all the methods. Because there were no significant differences in any metric across the thresholds, in the subsequent analysis, we focused on the average ICC across different thresholds. Second, we used paired sample *t*-tests to compare the ICC (pooled over all metrics) between methods, including contrasts between weighted and binary networks, the AAL90 atlas and Power264 atlas, and with and without GSR. Finally, we investigated the effect of the inter-scan interval on the TRT reliability of the graph-theoretical metrics. Because each subject was scanned three times within a week, we calculated pair-wise ICC between all pairs of visits: visit 1 and visit 2 (v1v2), visit 1 and visit 3 (v1v3), and visit 2 and visit 3 (v2v3). We then conducted a two-way repeated ANOVA on all the ICC, including a main effect of inter-scan intervals (three levels: v1v2, v1v3, v2v3), a main effect of modeling method (four levels: BG, binary network with global signal regression; BNG, binary network without global signal regression; WG, weighted network with global signal regression; WNG, weighted network without global signal regression), and an interaction effect between them. We also conducted a one-way repeated ANOVA using interval as the within-subject main effect (including post hoc *t*-tests) separately for each method. Additionally, we tested how individual characteristics affect TRT reliability by splitting the subjects based on biological sex (8 males and 8 females) and age (8 in the younger group and 8 in the older group, split by a median of 34.5). We calculated the ICC for each of the groups for all global metrics and across all methods and conducted paired sample t-tests between males and females, and younger and older subjects, respectively (each pair of samples had the same metric and method).

For nodal metrics, we performed a one-way ANOVA (plus post hoc pair-wise comparisons) on each metric (betweenness centrality, degree centrality, and nodal efficiency) with the main effect of the modeling method. We included all eight methods in the analysis. We also performed a paired *t*-test between all ICCs (pooled over all metrics) calculated with and without GSR.

## 3. Results

### 3.1. Test-Retest Reliability of Seven Global Metrics

**Figure 1:**
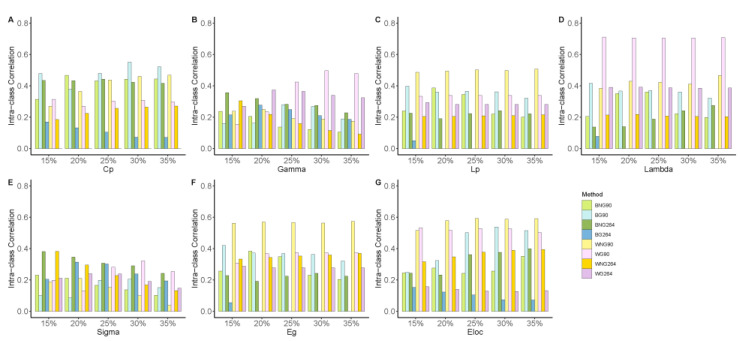
The ICC values for all seven-network metrics across different data processing and modeling strategies. Subfigures show the decomposition of ICC values across network density levels 15–35% for (**A**) Cp, (**B**) Gamma, (**C**) Lp, (**D**) Lambda, (**E**) Sigma, (**F**) Eg, and (**G**) Eloc, respectively. BG90: binary + global signal regression + AAL90, BNG90: binary + no global signal regression + AAL90, WG90: weighted + global signal regression + AAL90, WNG90: weighted + no global signal regression +AAL90, WG264: weighted + global signal regression + Power264, WNG264: weighted + no global signal regression + Power264; Cp: Clustering coefficient; Lp: Characteristic path length; Eg: Global efficiency; El: Local efficiency.

Figure 1 displays the ICC values for all seven-network metrics across different data processing and modeling strategies. In general, most global network metrics exhibited poor to fair TRT reliability (ICC: 0.32 ± 0.15, CV: 8.1% ± 6.0%). The least stable metric was small-worldness (sigma, ICC: 0.19 ± 0.07, CV: 9.9% ± 4.8%), and the most reliable metric was the normalized characteristic path length (Lambda, ICC: 0.39 ± 0.16, CV: 3.0% ± 1.6%). In addition, the ICC of the normalized characteristic path length reached its highest level (ICC: 0.71 ± 0.002, CV: 3.5% ± 1.3%) using the AAL90 atlas, weighted network modeling, and GSR.

Because the main effect of the threshold (one-way repeated ANOVA) within the range of 0.15–0.35 was not significant (F(1.70, 69.5) = 1.66, *p* = 0.20), in the following statistical analysis we adopted average ICC over thresholds as independent samples within the groups.

**Figure 2:**
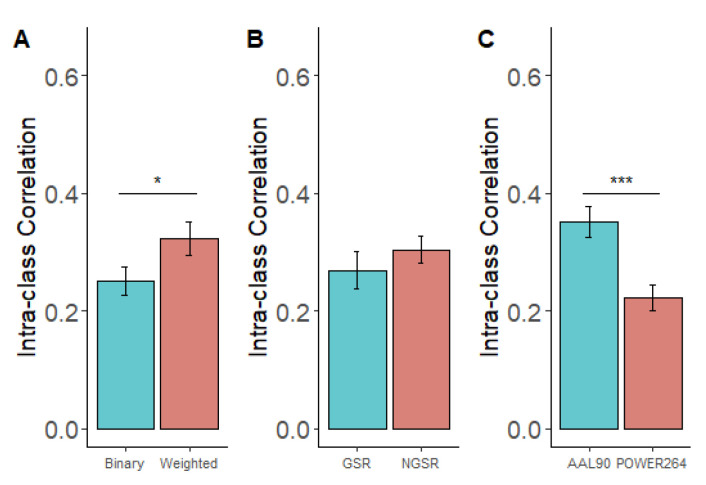
Comparisons of TRT reliabilities between three groups of methods. (**A**) Comparison of ICC values between the use of binary networks and weighted networks. (**B**) Comparison of ICC values between the use GSR or not (NGSR). (**C**) Comparison of ICC values between the use of AAL90 and Power264 atlases. *: *p* < 0.05, ***: *p* < 0.001.

To investigate the necessity of adopting GSR, weighted network, or Power264 atlas, we compared the mean ICC resulting from methods containing these elements. Paired sample *t*-tests (Figure 2) revealed that the reliability of weighted network metrics was significantly higher than that of binary network metrics (T(27) = 2.41, *p* = 0.022), and the use of the AAL90 atlas for brain parcellation provided higher ICC values than the use of the Power264 atlas (T(27) = 3.95, *p* = 0.001). However, GSR did not have a significant effect on the ICC values (T(27) = 1.15, *p* = 0.26).

**Figure 3:**
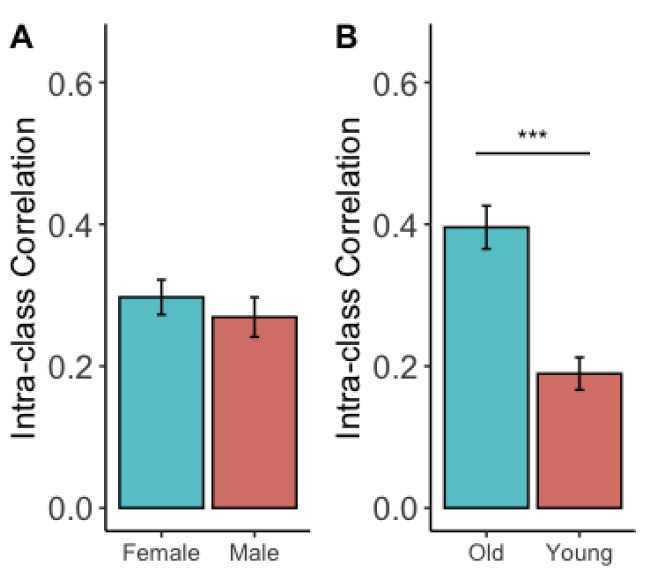
TRT reliabilities across different individual characteristics. (**A**) Comparison of the overall ICC values between males and females. (**B**) Comparison of the overall ICC values between the younger group and the older group. ***: *p* < 0.001.

We further assessed whether inherent individual characteristics affect the TRT reliability of the brain network metrics. We split our subjects into two types of groups based on their biological sex and age. Paired sample *t*-test (Figure 3) showed that sex did not have a significant impact on the overall ICC among brain metrics (ICC of male: 0.27 ± 0.21, ICC of female: 0.30 ± 0.18, T(55) = −0.68, *p* = 0.50), whereas age did have an impact, in which the older group had a significantly higher ICC than the younger group (younger group: age: 27.25 ± 3.95, ICC: 0.19 ± 0.17; older group: age: 43.63 ± 5.13, ICC: 0.40 ± 0.23, T(55) = −5.18, *p* < 0.0001).

### 3.2. Reliability of Two Visits

**Figure 4:**
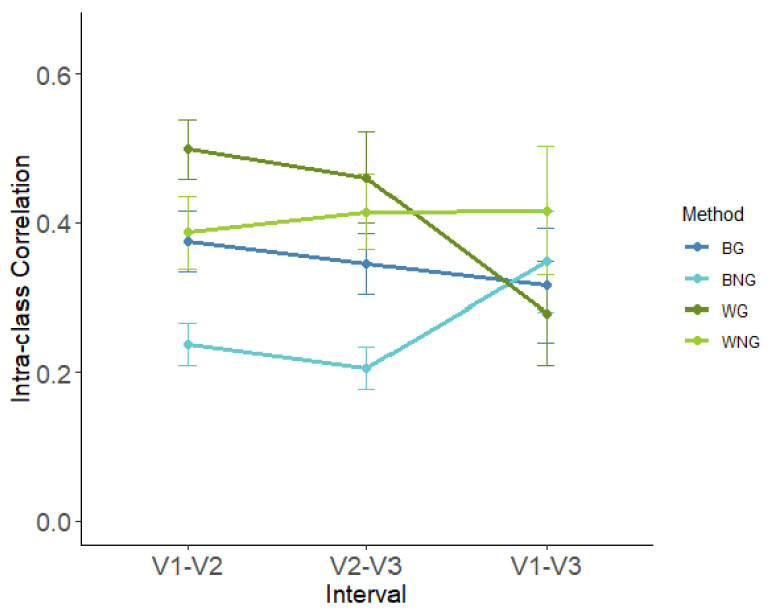
Average of test-retest reliability of global metrics as the function of interval. BG: binary + global signal regression, BNG: binary + no global signal regression, WG: weighted + global signal regression, WNG: weighted + no global signal regression. V1-V2: visit 1 (day 2) and visit 2 (day 3), V2-V3: visit 2 and visit 3 (day 5), and V1-V3: visit 1 and visit 3.

Using a two-way repeated ANOVA, we compared the TRT reliability between pairs of visits (i.e., different inter-scan intervals) across four methods (BG, BNG, WG, and WNG). Our results showed that there was not a significant main effect of inter-scan interval (F(1.15, 6.88) = 0.354, *p* = 0.60) or method (F(1.88, 11.29) = 3.32, *p* = 0.076), but a significant interaction effect (F(6, 36) = 4.66, *p* = 0.001) was observed between them. Figure 4 shows the relationship between TRT reliability and interval under four methods. WG yielded the highest value (ICC: 0.413 ± 0.059), while BNG yielded the lowest average ICC (ICC: 0.264 ± 0.032). 

To test the effect of interval on each method, we conducted a one-way repeated ANOVA using interval as the within-subject factor. The main effects of the interval were not significant for BG (F(1.1, 6.4) = 0.26, *p* = 0.77), BNG (F(1.1, 6.3) = 2.68, *p* = 0.15), and WNG (F(2, 12) = 0.174, *p* = 0.84), but they were significant for WG (F(2, 12) = 21.5, *p* < 0.001). For WG, post hoc *t*-tests (Bonferroni) revealed significantly lower ICC between visit1 and visit3 than that between visit1 and visit2 (*p* = 0.009) or between visit2 and visit3 (*p* < 0.001).

Table 2 shows the ICC of the seven metrics between three pairs of visits averaged over the threshold of 0.15–0.35. Given a certain method, the ICC of different metrics changed along the interval in different patterns. For example, the reliabilities of the normalized clustering coefficient (gamma) and small-worldness (sigma) decreased with an increase in the interval, regardless of the network construction methods. In addition, under the WG method, the ICC of all metrics, except the normalized characteristic path length (Lambda), decreased with a longer interval. 

### 3.3. Reliability of Three Nodal Metrics

**Figure 5:**
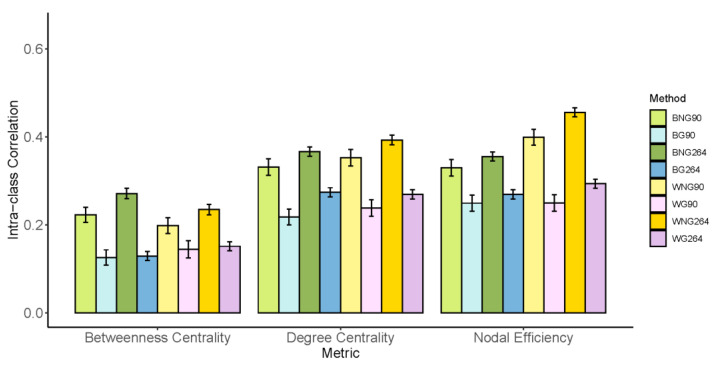
Test-retest reliability of nodal metrics across different data processing and modeling strategies. BG90: binary + global signal regression + AAL90, BNG90: binary + no global signal regression + AAL90, WG90: weighted + global signal regression + AAL90, WNG90: weighted + no global signal regression + AAL90, WG264: weighted + global signal regression + Power264, WNG264: weighted + no global signal regression + Power264.

The results of the reliability analysis for nodal metrics are shown in Figure 5. Generally, nodal efficiency has the highest TRT reliability (ICC: 0.33 ± 0.07, CV: 13.6% ± 5.5%), betweenness centrality has the least reliable nodal metric (ICC: 0.18 ± 0.05, CV: 106.5% ± 31.1%), and the reliability of degree centrality is in the middle (ICC: 0.31 ± 0.06, CV = 36.9% ± 12.3%).

Among all the modeling strategies, three of them resulted in higher reliabilities for each nodal metric compared with the rest of the strategies: BNG264, WNG90, and WNG264. In particular, the ICC of nodal efficiency under the WNG264 method was the highest (ICC: 0.45 ± 0.16, CV: 20.7% ± 5.3%). A one-way ANOVA showed significant differences in the TRT reliability across the eight modeling strategies for betweenness centrality (F(7, 1408) = 19.92, *p* < 0.001), degree centrality (F(7, 1408) = 22.98, *p* < 0.001), and nodal efficiency (F(7, 1408) = 36.07, *p* < 0.001). The results of the post hoc analysis (Bonferroni corrected) are shown in Supplementary Tables S1–S3. WNG264 yielded a significantly higher ICC than BG90, WG90, and WG264 for all three metrics, and its reliability was even significantly better than WNG90 for nodal efficiency. Considering that all the top three methods did not use GSR, we further combined the average nodal ICCs of all the metrics to test the influence of GSR per sec by a paired *t*-test, which proved that regressing out the global signal significantly decreases the nodal TRT reliability (△ICC: 0.11 ± 0.03, *p* < 0.001).

## 4. Discussion

According to previous studies, the major threats to the TRT reliability of rs-fMRI include scan conditions [56], physiological noise [57,58], data preprocessing, and network construction strategies [18,19,20,58]. In this study, we systematically investigated the test-retest reliability of the brain network topology based on strictly controlled rs-fMRI data across different preprocessing and modeling strategies. The overall reliability among the global network metrics was poor to moderate (0.000~0.592), except for the normalized characteristic path length (Lambda). The ICC of Lambda reached a good level (0.705~0.711) when we applied weighted connections and global signal removal. Similar results were reported by Wang et al., (2011), who found moderate reliability in Lambda, despite poor to low reliabilities in all other global metrics [20]. The TRT reliability of individual nodes had a large nodal variation (0.000~0.811), and the nodal efficiency (0.456) had the highest average ICC among the three metrics when we constructed weighted networks using the Power 264 atlas without global signal removal. 

One possible reason for the commonly found low ICC is that the ICC reflects the ratio of between-subject to within-subject variability. Since the functional connectivity of rs-fMRI across subjects could be highly homogeneous (i.e., small between-subject variability), the variation within the subjects was not small enough to yield a high ICC [59]. In support of this argument, higher reliability was found during the task state than the resting state due to the higher stability of event-related co-activations [60]. However, the reliability pattern depends on the content of the task; some tasks could improve the global ICC, whereas others could impair the global ICC [52]. In addition, despite a low ICC, the test-retest reliability could still be moderate to high [61]. Thus, it is important to also examine other criteria for TRT reliability. In this study, we also calculated the coefficient of variation. In contrast to the conclusion from ICC, the overall CV of global metrics was good (8.1% ± 6.0%), indicating small within-subject variations. Nevertheless, the CV of some nodal metrics, especially the betweenness centrality, was high (106.5% ± 31.1%), which was consistent with its poor ICC.

### 4.1. Factor Affecting the TRT Reliability of Global Metrics

For global metrics, comparisons between the methods revealed that the weighted network was generally more reliable than the binary network. One crucial aspect of the weighted network is that it preserves more detailed information on connectivity strength. As a result, the weighted network can detect subtle changes in connectivity, and this complexity leads to high resistance to external disturbances. Many other studies have also focused on this issue, and one review [18] reported a slight advantage of weighted methods by analyzing data from many previous studies [19,20,62,63,64,65]. Nevertheless, most studies today still prefer binary networks, partly due to their simplicity of interpretation.

Removing the global signal showed a slight but non-significant disadvantage on the global metrics. Similar results have been reported by Andellini et al., (2015). Even though GSR had a significant negative effect on the reliability of the clustering coefficient, the overall effect across metrics was not significant [18]. In another study, the reliability of Lambda even increased after GSR [19]. Future research needs to investigate this issue with a broader sample size and should potentially address the effect of GSR while varying other steps during the network construction.

In addition to the major findings regarding data processing and modeling strategies, we also examined whether individual demographic characteristics (sex and age) affected the TRT reliability of network global metrics. While we did not find significant differences in ICC between males and females, we found that subjects in the older group (35~50 years) had higher ICC than those in the younger group (22~34 years). Previous studies comparing the ICC of BOLD fMRI across age groups suggested an inverse U-shaped relationship as follows: the ICC is lower during infancy and childhood, peaks in adulthood with the maturation of the brain, and decreases in older adults [66,67,68]. However, such evidence is scarce, and it is not clear which age range has the highest ICC. Our results, although preliminary and limited in sample size (N = 8 for each group), can provide a finer characterization of the relationship.

### 4.2. The Effect of Inter-Scan Interval on TRT Reliability

For TRT reliability between pairs of visits, we only found subtle but not significant differences. In other words, the overall TRT reliability of global metrics remained stable during the entire study and was independent of the passage of time. However, such robustness to inter-scan intervals relied on specific data processing strategies. For instance, the reliability of metrics under WG decreased when the inter-scan intervals were longer. The source of variability in longer intervals may be the fact that participants kept adjusting their lifestyles and biological clocks in the new environment. Among all metrics, longer intervals mostly affected the reliability of Gamma and Sigma. 

### 4.3. TRT Reliability of Nodal Metrics

All three nodal metrics exhibited poor to low reliabilities on average, yet the degree centrality and nodal efficiency were more reliable than the betweenness centrality, consistent with the findings of Du et al., (2015) [45]. These results could be explained by the definitions of these metrics; the degree centrality and nodal efficiency of a node only depend on direct connections with it, whereas betweenness centrality is calculated by the connections of adjacent nodes. As a result, connectivity changes in a remote node will have an impact on the betweenness centrality of the current node, but not on the degree centrality or nodal efficiency. Therefore, the reliability of betweenness centrality is in general low.

What is evident from the comparison of methods is that applying GSR significantly lowers the TRT reliability for both binary and weighted networks and with both parcellations. This is inconsistent with Du et al., (2015), who detected slight but not significant disadvantages of the GSR [45]. Such a difference may be due to the different network sizes in the two studies; in Du et al., (2015), the networks were based on 25,218 voxels that were much larger than those in the current study [45]. Therefore, the benefit of GSR in improving sensitivity offsets the loss of reliability in much denser networks.

### 4.4. Opposite Effects of Parcellations on Global and Nodal Metrics

It is worth noting that the effect of parcellations on the global metrics was opposite to that on the nodal metrics: the AAL-90 atlas generated higher reliabilities for global metrics than the Power264 atlas, whereas the optimal processing and modeling strategy for nodal network metrics was the one that applied the Power264 atlas (i.e., WNG264). There are many differences between these two atlases. The AAL-90 defines 90 brain regions based on anatomical features, and each ROI encompasses a wide range of brain tissues, whereas the Power264 atlas defines 264 spherical ROIs based on functional co-activations, and each ROI is a spherical region containing a fixed and limited number of voxels. Therefore, on a global scale, ROIs from the AAL-90 atlas always come from the same inherent anatomical organizations, independent of the functional state of the brain, and produce a more robust pattern of connectivity in general. On the other hand, on a nodal scale, the Power264 atlas has higher spatial resolutions, and its use of smaller ROIs reduces the regional inhomogeneity of the ROI [69]. For a given local property, the reliability of a node can be less affected by the signals of its surrounding areas. 

### 4.5. TRT Reliability of BOLD fMRI Compared to Other Modalities

In addition, the reliability of BOLD fMRI may become a disadvantage compared with other modalities. For example, cerebral blood flow (CBF) quantified by arterial spin-labeled (ASL) perfusion MRI couples with regional brain activity, perfusion, and metabolism [70], which also serve as biomarkers in clinical settings. While the common ICC of resting-state BOLD fMRI is poor to moderate (0.2~0.6) [20,27,45,46,71], that of CBF is usually greater than 0.6, falling in the good to excellent range [72,73,74]. In our previous work, we evaluated the TRT of resting-state and task-based absolute CBF, as well as task-induced relative CBF, and found that ICC values ranged from good to excellent (ICC > 0.6) for absolute CBF and poor for relative CBF (ICC < 0.4) [75]. On average, absolute CBF, rather than relative CBF, has a better TRT than the small world network properties based on rs-fMRI.

### 4.6. Limitations

Our study has several limitations. The first limitation is the choice of parcellation schemes. In the current comparison, we only included two representative parcellations: AAL-90 and Power264. To better understand the impact of parcellation, future studies should include more atlases in the comparison with different numbers of subdivisions and different parcellation algorithms. A fine-grained strategy, such as the voxel-wise analysis, should also be considered. For example, previous studies have reported overall good to high ICCs with voxel-wise network construction [76]. 

Another limitation is the limited range of the inter-scan interval. An eligible biomarker should remain stable in the long term in the absence of a disease. In our study, the longest interval was three days; thus, it was difficult to test such eligibility directly. Instead, we only examined the performance of graph theoretical metrics after a short-term manipulation and tried to predict the long-term effects. 

Finally, in this initial study, we were only able to test a small sample of 16 control subjects in a very strictly controlled in-laboratory 5-day and 4-night study. Although the use of strict controls added to the methodological strength of the study to help rule out potential confounders during the data collection, a potential drawback is that it is unclear whether our findings can be generalized to most other studies that do not have such a stringent design. Future replications with larger sample sizes and different data collection protocols are needed to test the generalizability of the current findings. 

## 5. Conclusions

In summary, in the existing literature, as of now we are the first to comprehensively investigate the influence of data processing and modeling strategies on the TRT reliability of both global- and nodal-level graph-theoretical metrics while strictly controlling the subjects’ behaviors. By strictly monitoring the daily activities of our subjects in the laboratory for five days, we attenuated the impact of external factors on brain activities.

Several important suggestions can be derived from our findings for the implications of the future utility of graph theoretical modeling in brain network analyses. First, when using a global network metric as a clinical biomarker, the normalized characteristic path length is highly recommended. Based on our results, it has the highest ICC among all the global metrics, especially when calculated using weighted networks and global signal regression. In terms of general methods, researchers should consider using weighted networks instead of binary networks and using AAL-90 instead of the Power264 atlas, as they both showed significantly higher ICC than their counterparts. Researchers should be cautious when applying global signal removal. Similarly to previous studies, we did not find a clear advantage of including or excluding GSR in the resulting ICC. When using a nodal network metric, we recommend degree centrality and nodal efficiency, as both have consistently better TRT reliability than betweenness centrality. Finally, the reliability of brain network metrics may decline longitudinally, which is a threat to experiments with long scan intervals.

## Figures and Tables

**Table 1:** Summary of eight data processing methods.

	BG90	BNG90	WG90	WNG90	BG264	BNG264	WG264	WNG264
Network type	Binary	Binary	Weighted	Weighted	Binary	Binary	Weighted	Weighted
Global signal regression	Yes	No	Yes	No	Yes	No	Yes	No
Parcellation	AAL90	AAL90	AAL90	AAL90	Power264	Power264	Power264	Power264

Note: BG90: binary + global signal regression + AAL90, BNG90: binary + no global signal regression + AAL90, WG90: weighted + global signal regression + AAL90, BG264: binary + global signal regression + Power264, BNG264: binary + no global signal regression + Power264, WNG90: weighted + no global signal regression + AAL90, WG264: weighted + global signal regression + Power264, WNG264: weighted + no global signal regression + Power264.

**Table 2:** ICC between pairs of visits.

	BG			BNG			WG			WNG		
	v1v2	v2v3	v1v3	v1v2	v2v3	v1v3	v1v2	v2v3	v1v3	v1v2	v2v3	v1v3
Cp	0.519	0.521	0.427	0.416	0.317	0.557	0.463	0.302	0.176	0.324	0.332	0.543
Gamma	0.477	0.262	0.000	0.209	0.247	0.066	0.516	0.389	0.202	0.217	0.299	0.127
Lambda	0.253	0.317	0.473	0.159	0.120	0.474	0.662	0.788	0.670	0.548	0.449	0.336
Lp	0.250	0.312	0.465	0.213	0.127	0.455	0.480	0.401	0.158	0.476	0.408	0.589
Sigma	0.435	0.198	0.000	0.208	0.226	0.096	0.295	0.308	0.132	0.201	0.220	0.057
Eg	0.258	0.321	0.471	0.231	0.130	0.460	0.487	0.449	0.188	0.492	0.606	0.593
Elocal	0.434	0.485	0.373	0.231	0.274	0.330	0.586	0.589	0.426	0.449	0.584	0.668

Note: v1: The first visit on day 2; v2: the second visit on day 3; v3: the third visit on day 5; BG: binary network with global signal regression; BNG: binary network without global signal regression; WG: weighted network with global signal regression; WNG: weighted network without global signal regression.

## Data Availability

The original contributions presented in the study are included in the article. Further inquiries can be directed to the corresponding authors.

## Supplementary Materials

The following supporting information can be downloaded at: https://www.mdpi.com/article/10.3390/brainsci13050825/s1, Table S1: Pair-wise differences of the betweenness centrality ICC among eight strategies. Table S2: Pair-wise differences of the degree centrality ICC among eight strategies. Table S3: Pair-wise differences of the nodal efficiency ICC among eight strategies.
