# Supplementary material for: Test-Retest Reliability of Resting Brain Small-World Network Properties across Different Data Processing and Modeling Strategies
Source: Brain Sci. 2023 May 19;13(5):825. doi: 10.3390/brainsci13050825 (PMC10216465; doi:10.3390/brainsci13050825)
Supplement: Supplementary file 1 [file brainsci-13-00825-s001.zip › brainsci-2292737-supplementary.pdf]

Table S1

Pair-wise differences of the betweenness centrality ICC among eight strategies.

Betweenness Centrality	BNG90	BG90	WNG90	WG90	BNG264	BG264	WNG264	WG264
BNG90								
BG90	0.097**							
WNG90	0.025	-0.072						
WG90	0.079*	-0.018	0.054					
BNG264	-0.048	-0.145***	-0.073*	-0.127***				
BG264	0.094***	-0.003	0.069*	0.015	0.142***			
WNG264	-0.012	-0.109***	-0.037	-0.091***	0.036	-0.106***		
WG264	0.071*	-0.026	0.0467	-0.007	0.120***	-0.022	0.083***	

Note: BNG90: binary + no global signal regression + AAL90, BG90: binary + global signal regression + AAL90, WG90: weighted + global signal regression + AAL90, BNG264: binary + no global signal regression + POWER264, BG264: binary + global signal regression + POWER264, WNG90: weighted + no global signal regression +AAL90, WG264: weighted + global signal regression + POWER264, WNG264: weighted + no global signal regression + POWER264. *: $p<0.05$, **: $p<0.01$, ***: $p<0.001$, Bonferroni corrected.

Table S2

Pair-wise differences of the degree centrality ICC among eight strategies.

Degree Centrality	BNG90	BG90	WNG90	WG90	BNG264	BG264	WNG264	WG264
BNG90								
BG90	0.114***							
WNG90	-0.021	-0.134***						
WG90	0.093**	-0.020	0.114***					
BNG264	-0.035	-0.148***	-0.014	-0.128***				
BG264	0.058	-0.056	0.078**	-0.036	0.092***			
WNG264	-0.061	-0.175***	-0.041	-0.155***	-0.027	-0.119***		
WG264	0.062	-0.051	0.083**	-0.031	0.097***	0.005	0.123***	

Note: BNG90: binary + no global signal regression + AAL90, BG90: binary + global signal regression + AAL90, WG90: weighted + global signal regression + AAL90, BNG264: binary + no global signal regression + POWER264, BG264: binary + global signal regression + POWER264, WNG90: weighted + no global signal regression +AAL90, WG264: weighted + global signal regression + POWER264, WNG264: weighted + no global signal regression + POWER264. *: $p<0.05$, **: $p<0.01$, ***: $p<0.001$, Bonferroni corrected.

Table S3

Pair-wise differences of the nodal efficiency ICC among eight strategies.

Nodal Efficiency	BNG90	BG90	WNG90	WG90	BNG264	BG264	WNG264	WG264
BNG90								
BG90	0.080*							
WNG90	-0.070	-0.150***						
WG90	0.079*	-0.001	0.149***					
BNG264	-0.026	-0.106***	0.044	-0.105***				
BG264	0.060	-0.020	0.129***	-0.019	0.086***			
WNG264	-0.126***	-0.206***	-0.057	-0.206***	-0.101***	-0.186***		
WG264	0.036	-0.044	0.105***	-0.044	0.062**	-0.024	0.162***	
